# Vulcanite Combinations

**Published:** 1870-06

**Authors:** W. Geo. Beers

**Affiliations:** Montreal.


### ARTICLE VII.

### *Vulcanite Combinations.*

By W. Geo. Beers, Montreal.

The exclusive use of either red, black or pink rubber, as a base for artificial teeth, have their separate objections. Certain objections to the red may be removed by the use of the brown or black, but the color of these is a strong argument against their use with many patients. The pink compounds would seem to fill the void; but all the light shades of vulcanite are inferior in strength and durability to the red or black, having a much smaller percentage of caoutchouc, and a larger amount of earthy matters or metallic oxides, used to tone down the original color. In 100 parts of the best English pink there is a percentage of 60 parts of fixed matter, while in the best red and black there is, in 100 parts, only from 3 to 6 of this objectionable foreign matter. The consequence is, that the worst colors are the best for all purposes. The following is the method I use for obviating the separate objections referred to, while combining any separate excellence they possess.

Put a sheet of red or black rubber in boiling hot water, and when softened, pass it through rolling mills, until it is reduced to half its usual thickness. It is better to cut the sheet in two longitudinal strips previous to rolling, as the rolling widens the sheet, and it is liable to catch and tear at the sides. If for instance, the case is an upper set, make a paper pattern of the palatine surface of the model, keeping at least a quarter of an inch from the pivots of the teeth, and the back part where the plate is to terminate. Cut the red or black rubber to correspond with this pattern, and place it on thə palatine surface of the model, over the air chamber. Now take strips of pink rubber, and pack them regularly under the pivots as the teeth lie in the flask. Cut a piece of pink rubber a quarter of an inch wider in circumference than the red piece, and place it properly in the part of the flask containing the teeth. Pack red rubber around the pivots, and sufficient pink elsewhere to prevent red oozing through, and wherever it is necessary. The result, of course is that you have red vulcanite for strength on the upper palatine surface where it does not show, and pink on the lower or visible surface. Nothing makes a handsomer "rubber" set than the two layers combined. In preparing the set for the flask, I always alter the wax to come up high to the back of the crowns of the teeth so that the depression thereby caused in the plaster, will accomodate sufficient pink. rubber to prevent any red passing through. The wax model should be smoothened as well as possible, and every precaution taken to avoid much use of the bur when finishing.

I think the above method is better tor all purposes than the entire use of pink rubber, as it gives the upper palatine surface of the plate the most durable, and the lower surface the most beautiful.—*Canada Journal of Dental Science.*

---

